# Synthesis of Highly Active Pd@Cu–Pt/C Methanol Oxidation Electrocatalysts via Continuous, Co-Electroless Deposition

**DOI:** 10.3390/nano11030793

**Published:** 2021-03-19

**Authors:** Gregory L. Tate, Bahareh Alsadat Tavakoli Mehrabadi, Wen Xiong, Adam Kenvin, John R. Monnier

**Affiliations:** 1 Department of Chemical Engineering, University of South Carolina, Columbia, SC 29208, USA; gltate@email.sc.edu (G.L.T.); bahareh.mehrabadi@nikolamotors.com (B.A.T.M.); wxiong@email.sc.edu (W.X.); akenvin@email.sc.edu (A.K.); 2 Research and Development, Nikola Motors, Phoenix, AZ 85040, USA

**Keywords:** bifunctional catalyst, electroless deposition, fuel cells, heterogeneous catalysis, preparation

## Abstract

Controlled deposition of metals is essential for the creation of bimetallic catalysts having predictable composition and character. Continuous co-electroless deposition (co-ED) permits the creation of bimetallic catalysts with predictive control over composition. This method was applied to create a suite of Cu–Pt mixed-metal shell catalysts for use in methanol electrooxidation in direct methanol fuel cell applications (DMFCs). Enhanced performance of Cu–Pt compositions over Pt alone was predicted by existing computational studies in the literature. Experimental evidence from this study supports the bifunctional catalyst explanation for enhanced activity and confirms the optimum Cu:Pt ratio as Cu_3_Pt for this methanol electrooxidation. This ability to control the composition of a bimetallic shell can be extended to other systems where the ratio of two metals is critical for catalytic performance.

## 1. Introduction

The move toward more environmentally sound transportation and portable power devices has been an important focus of research in recent years. Fuel cells have provided an alternative way to supply power since the 1960s [1]. However, there are bottlenecks, one being the low-energy density of hydrogen (10.8 MJ/m^3^ at STP) and the challenges of storing, metering, and transporting a highly compressed and flammable gas [2]. A promising alternative technology is to replace H_2_ with methanol as a hydrogen source. Methanol is much more energy-dense (18.1 GJ/m^3^ at STP) than H_2_ and exists as a “drop-in” fuel [2]. Existing gasoline metering and distribution can be converted to methanol without much difficulty.

Direct methanol fuel cells (DMFCs), however, also have limitations regarding their activity. The oxidation of methanol produces CO as an intermediate, and Pt strongly adsorbs CO. Thus, CO inhibits further MeOH oxidation placing an upper limit on the rate of MeOH oxidation [3,4].

There are three reactions that take place during methanol electrooxidation. The first reaction is the adsorption of methanol on Pt, where it decomposes into CO, donating 4e^−^ to the electrode and liberating 4H^+^ (Equation (1)). The second reaction is the decomposition of water on Pt to give OH and H^+^ and one e^−^ (Equation (2)). The final reaction (Equation (3)) is the oxidation of CO with adsorbed OH to form CO_2_, which easily desorbs to complete the catalytic cycle [3,4,5,6,7]:

CH_3_OH + Pt → Pt–CO + 4H^+^ + 4e^−^ (1)

H_2_O + Pt → Pt–OH + H^+^ + e^−^ (2)

Pt–OH + Pt–CO → CO_2_ + H^+^ + 2Pt + e^−^ (3)

At most operating conditions, the rate-determining step is generally accepted to be the oxidation of adsorbed CO. One of the methods to lower the inhibition effect of CO involves the addition of a second metal proximal to the active Pt component. Proper placement of the second metal can alter the electronic structure of Pt or provide a different catalytic site to introduce bifunctionality. It has been experimentally shown that the d-band of Pt can be altered by either lattice compression or expansion, which changes the adsorption strength of CO on Pt [8,9]. Bifunctionality involves the addition of a second metal in close proximity to Pt that functions as an adsorption site for the decomposition of H_2_O to give OH_ads,_ which can react with Pt-CO to form CO_2_ by reaction (3) [5,10,11,12]. This bifunctional mechanism is believed to be responsible for the enhanced activity of the Ru–Pt system for methanol electrooxidation [11]. Indeed, the addition of ruthenium to platinum supported on carbon using electroless deposition methods has been shown [12,13] to be an effective method of dramatically increasing DMFC activity, corroborating the bifunctional mechanism proposed earlier by Watanabe [10,11,12], among others.

The key is to be able to control the amounts and locations of the two metal components to give the optimal effect. Typically, the most common method is co-impregnation, co-precipitation, or co-reduction of both metal salts on the carbon support [14]. With these procedures, there is no assurance that bimetallic particles are consistently formed or that the bimetallic particles have uniform compositions. Thus, analyses and performances of materials are based on average bulk compositions instead of true metal ratios at the bimetallic particle scale. To solve this, electroless deposition (ED) has been used to selectively deposit the second metal only on the surface of a pre-existing primary metal by catalytic activation of a reducing agent on the primary metal surface [10,13,15,16,17,18,19,20]. This process ensures that the secondary metal is deposited only on the primary metal and not on the support [15]. For example, in earlier work [12], bimetallic catalysts with different monolayer (ML) coverages of Ru on 20 wt % Pt/C were prepared by ED and evaluated for methanol electrooxidation. The Pt mass activity (425A/g_Pt_) of the sample with 0.5 ML Ru coverage (1.1 wt % Ru—surface Pt:Ru = 1:1) was seven times higher than a 20 wt % Pt/C catalyst and 3.5 times higher than a commercial catalyst with a 1:1 Pt: Ru bulk atomic ratio of 6.8 wt % Ru—13.2 wt % Pt/C. Additionally, the amount of Ru required for the highest activity was only 1.1 wt % Ru, compared to 6.8 wt % Ru for the commercial catalyst. The ability of ED to target Ru only on the Pt surface dramatically lowered Ru requirements.

In this study, we use electroless deposition to prepare Cu–Pt catalysts for methanol electrooxidation. However, rather than depositing Cu on a Pt surface, both Cu and Pt have been simultaneously co-deposited on a different core metal particle to create a mixed Pt–Cu shell on a primary metal core. In this process, two metal salts are simultaneously added along with a reducing agent to an ED bath containing a 5 wt % Pd/C base catalyst. The relative ratios of metal deposition are controlled by the intrinsic reduction potentials of the metal salts and the concentrations and pumping rates of the Cu and Pt salts. The reasons for codeposition are to prepare a more uniformly distributed, bimetallic layer to maximize bifunctionality and possibly improve stability. Further, it should be possible to use minimal amounts of each of the two metals to reduce the cost of synthesis. While this is not critical for Cu, it is clearly an important consideration for the much more expensive Pt component.

Cu–Pt alloys were selected because they have been studied both experimentally and computationally for MeOH electrooxidation and represent a good case for comparison. One of the benefits of using ED for the deposition of either single metals or deposition of two metals is the general versatility of this method. Experimental studies have shown increased activity for Cu–Pt alloys relative to either Pt or Cu catalysts [5]. It is also known that Cu dissociates H_2_O and does not adsorb CO, and should provide a bifunctional site for OH_ads_ [21]. Additionally, computational literature has predicted that several Pt–X (X = Cu, Ru, Sn) alloys could provide lower overpotential for methanol electrooxidation compared to Pt alone [5]. Rossmeisl and Tritsaris [6] calculated that a Cu_3_Pt surface moiety offers the lowest overpotential for MeOH electrooxidation. Since continuous codeposition should permit very good control over the shell composition, Cu–Pt shells have been prepared over a range of Cu and Pt ratios to determine the optimum catalyst composition and whether experimental results are corroborated with computational predictions.

Any metal with sufficient ability to oxidize a reducing agent can be selected as a core metal, but for these studies, a commercial 5 wt % Pd/C (dispersion = 21.6%, average particle diameter = 5.2 nm) was selected as the core metal base catalyst (Engelhard 5 wt % Pd/CP56). Although the existence of ternary Pt–Cu–Pd alloys is argued in the literature, the low-temperature aqueous method of preparation is thought to preclude any bulk ternary alloy formation [22,23,24,25]. There is insufficient thermal energy to force alloying of the Cu–Pt shell with the bulk Pd core at room temperature. It is presumed that the shell contains Cu and Pt alone. Furthermore, of importance, palladium is known to be inactive for methanol electrooxidation under acidic conditions [26,27,28,29], ensuring that activity was only from the Pt–Cu shell and not the Pd core.

## 2. Materials and Methods

### 2.1. Catalyst Preparation

A series of Pt–Cu/Pd/C catalysts was prepared by continuous co-ED following the general procedures discussed in prior work [30]. The core catalyst on which the mixed-metal shell is deposited is commercial 5 wt % Pd/C (disp = 21.6% D = 5.2 nm) was used as received. In this case, a master 300 ppm PtCl_6_^2−^ solution was prepared. To this solution, a 5:1 molar equivalent of ethylenediamine (EN) as stabilizing agent to prevent the unwanted thermal reduction of PtCl_6_^2−^ (with the reducing agent) was added to the volume of the PtCl_6_^2−^ solution to be loaded into the syringe preceding the ED experiment. A 500 ppm copper solution was prepared fresh daily from Cu(NO_3_)_2_·6H_2_O. No complexing agent was added to this solution because of the lower reduction potential of Cu^2+^ compared to PtCl_6_^2−^ (E**°** = 0.34 V and E**°** = 0.72 V, respectively). Reducing agent selection was based on the work of Ohno [31] and Djokić [32]. Dilute aqueous hydrazine was selected as the reducing agent because of its facile oxidation over Pd, Pt, and Cu at slightly alkaline conditions and the “clean” nature of oxidation (N_2_H_4_ oxidizes into H_2_O and N_2_, whereas other reducing agents can leave decomposition products that can affect the ED process.) The amount of N_2_H_4_ was selected to be in excess of the electron requirements for all metal salts by a factor of 5:1 because the inherent instability of N_2_H_4_ results in H_2_ evolution to give inefficient use during ED. The overall equations for continuous co-ED are listed as Equations (4)–(6) as proposed by Djokić [32]. N_2_H_4_ is initially adsorbed and dissociated on the Pd core to give four adsorbed H species, which in turn reduce Pt(EN)*_x_*Cl_6_^2−^ and Cu^2+^ leaving Pt^0^ and Cu^0^ on the surface (Equations (5) and (6)). As the reaction proceeds, N_2_H_4_ can be readily adsorbed and oxidized on deposited Pt^0^ and Cu^0^, providing additional locations for Cu or Pt salt reduction. The values of the oxidation and reduction potentials for each of the reactants indicate ED should readily occur:

N_2_H_4_ + Pd^0^ → N_2_ + 4H_ads_ (basic) E° = +1.16 V (4)

Pt(EN)*_x_*Cl_6_^2−^ + 4OH^−^ + 4H_ads_ → Pt^0^ + xEN + 6Cl^−^ + 4H_2_O E° = +0.72 V (5)

Cu^2+^ + 2OH^−^ + 2H_ads_ → Cu^0^ + 2H_2_O E° = +0.34 V (6)

**Figure 1:**
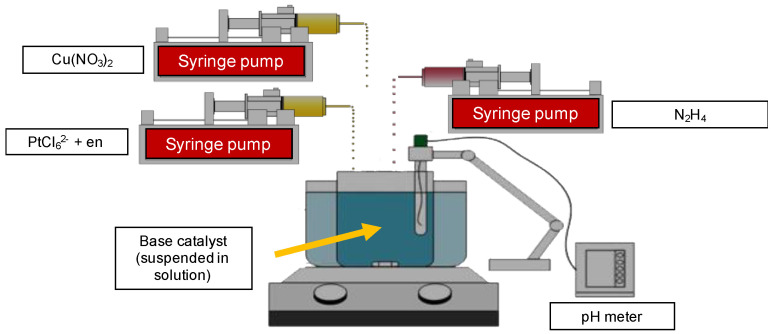
Image of the experimental setup for continuous co-electroless deposition (co-ED). New Era Pump Systems NE-300 pumps were used to add reagents during ED. A pH meter continuously monitored the pH of the ED bath. Bath temperature was maintained (in this case at 25 °C) but could be controlled up to 90 °C using a digital, PID heated stirrer to regulate temperature and stir the bath.

To perform continuous co-ED, 500 mg of base catalyst was added to DI water in a disposable plastic beaker and pH was adjusted to 9 using a NaOH solution. All three microcontroller-driven syringe pumps were started simultaneously, and the molar rate of pumping was controlled by the concentration of the solution and individual pumping rates to give the desired ratios of Pt and Cu deposited on the core metal. A schematic of the experimental setup is shown in Figure 1. Three microcontroller-driven syringe pumps (New Era Pump Systems, NE-300, Farmingdale, NY, USA) were used to add each reagent separately at set rates (Cu salt, Pt salt, and N_2_H_4_). The ED bath was continually stirred, and pH was monitored throughout the reaction. The pH of the solution was adjusted by 0.1 M NaOH or HCl, if necessary, throughout the course of the experiment to maintain pH = 9. In all cases, a combined Cu and Pt shell of five theoretical monolayers was targeted, although the actual coverage of the bimetallic shell depended on the actual amount of metal deposited. Active Pd surface area site concentration used for theoretical ML calculations was determined from H_2_–O_2_ titration (AutoChem II 2920 Chemisorption analyzer, Micromeritics, Norcross, GA, USA) of the Pd core catalyst. Aliquots (0.5 mL) of the ED bath were taken at preselected time intervals during the course of the experiment to determine the number of metal salts remaining in the solution (and by difference, the number of metals deposited on the base catalyst). After the deposition time was completed, the catalyst was vacuum filtered and rinsed with a large excess of DI water to remove unreduced metal ions, EN, and N_2_H_4_ (not likely to be present due to instability and non-selective evolution of H_2_.) Aliquots were immediately analyzed by AAS (PerkinElmer AAnalyst 200, Waltham, MA, USA). All samples were dried overnight in flowing air at 25 °C and then stored in capped bottles.

For comparison, a commercial 20 wt % Pt/XC72 sample was evaluated for methanol electrooxidation. This catalyst was evaluated as-received and characterized by STEM, XRD, and H_2_–O_2_ titration to give average diameter d: d(STEM) = 3 ± 1 nm, d(XRD) = 3.2 nm, and d(H_2_–O_2_ titration) = 6.1 ± 0.3 nm.

### 2.2. Characterization

Dried samples were analyzed by powder XRD (Rigaku Miniflex with D/tex Ultra 250 1D silicon strip detector, Tokyo, Japan) to determine whether Pt or Cu oxides were present. If bulk Pt or Cu oxides were present, it would suggest that uniform deposition did not occur during ED. Bulk Cu^0^ particles at the nm scale would likely undergo facile oxidation to Cu_2_O, while deposition of a mixed alloy Pt–Cu shell should inhibit the formation of a bulk Cu_2_O phase. Further, the presence of crystalline Pt^0^ and Cu^0^ phases would also indicate segregation of the two components during co-ED.

Additionally, to quantify Pt site concentrations at the surface of the shell, H_2_–O_2_ titration (AutoChem II 2920 Chemisorption analyzer, Micromeritics, Norcross, GA, USA) was used to characterize all samples. All samples were exposed to a flowing stream of H_2_ for 1 h at 45 °C. Following this, the sample was swept with flowing Ar for 1 h at the same temperature to remove any physically adsorbed H_2_. The sample was next pre-covered with oxygen by flowing in 10% O_2_/balance He for 30 min, and residual O_2_ was removed by flowing in Ar for 30 min. Titration of the surface was done with 10% H_2_/balance Ar pulses, dosed until the peak area did not change [33,34]. Uptake was determined by the summation of pulse area and gas loop volume. This procedure was repeated three times per sample to ensure reproducibility, with average values and standard deviation reported.

STEM imaging was performed on select samples to determine catalyst morphology and ensure core-shell structure with mixed Cu–Pt shell. A JEOL 2100 F 200 kV scanning transmission electron microscope(Tokyo, Japan) equipped with a CEOS Cs-corrector illumination source and Fischione Model 3000 high angle annular dark-field (HAADF, Export, PA, USA) detector was used for imaging. Minimization of line noise was accomplished by synchronized 60 Hz scanning, with 15.8 µs pixel dwell time.

No pretreatment, other than drying at room temperature in flowing air, was used before any characterization procedure.

### 2.3. Cyclic Voltammetry

Cyclic voltammetry (CV) procedures are largely repeated from previous work with a different catalyst system studying the same reaction [12]. Cyclic voltammetry studies were performed using a 5 mm diameter Pt disk coated with catalyst as the working electrode with a bare Pt wire used as the counter electrode. For the reference electrode, a Luggin capillary Hg/Hg_2_SO_4_ electrode was employed. To coat the working electrode, an ink was prepared by sonicating 10 mg of dry catalyst in a 10 mL solution consisting of a 1:1 volumetric ratio of isopropyl alcohol (IPA) and DI water, the electrode ink having a final catalyst concentration to solvent ratio of 1 g/L. To coat the working electrode, an 18.5 μL sample of the ink was added dropwise to the Pt disk. To secure the ink to the surface of the electrode, a 5 μL solution of 5 wt % Nafion: IPA was added atop the ink.

CV analyses were performed using an N_2_-purged bath of 0.5 M H_2_SO_4_ and 1 M MeOH; all evaluations were performed at 25 °C. At bath conditions, the reference electrode had a potential of 0.682 V versus the standard hydrogen electrode (SHE) and all potentials reported are referenced to SHE value [35]. All catalysts were conditioned for 50 cycles before CV measurements at a rate of 50 mV/s from 0–1.2 V. CV measurements were performed from 0–1.2 V at a rate of 5 mV/s and repeated three times to ensure reproducibility. The average forward peak current (I_f_) at ~0.85 V from these three trials was used to determine reported methanol electrooxidation activity.

## 3. Results

### 3.1. Preparation/Synthesis

**Figure 2:**
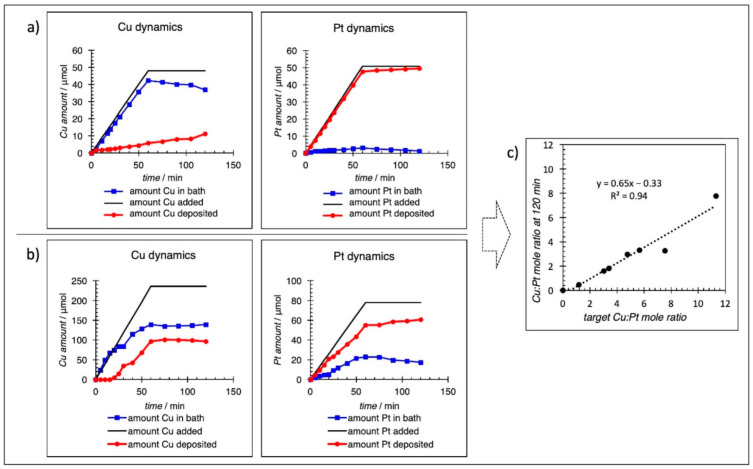
Deposition kinetics for a feed ratio of (**a**) 1:1 = Cu:Pt (**b**) 3:1 = Cu:Pt. The solid line indicates the total amount of metal added, the red line indicates metal deposited in the mixed shell, and the blue line indicates the metal salt remaining in the bath. Final shell composition was (**a**) Cu:Pt = 0.23:1 and (**b**) Cu:Pt = 1.6:1. Results from deposition kinetics shown for all materials made plotted (**c**) where the amount of Cu:Pt added by a syringe pump (target) is compared to the amount (Cu:Pt) actually deposited after 120 min. Linear regression is shown by the dashed line.

The kinetics for two examples of co-electroless deposition are shown in Figure 2a,b. Figure 2a shows Cu and Pt deposition for the addition of equal molar concentrations of PtCl_6_^2−^ and Cu^2+^ over a 60 min time interval. The straight-line deposition rates over the full time interval indicate the formation of a shell with constant bimetallic composition. As stated earlier, a hydrazine solution at 5× molar excess was added from a third syringe. Approximately 50 μmol of each salt was added over a 60 min period, and then the bath was left in a batch mode for an additional 60 min. The results clearly show that PtCl_6_^2−^ is reduced much more rapidly than Cu^2+^, possibly because of the higher reduction potential of PtCl_6_^2−^ compared to Cu^2+^, although kinetics are favorable for both salts. From 60–120 min, there is a small amount of additional Cu deposition, indicating some residual N_2_H_4_ is left in the bath. The final composition of the bimetallic shell was Cu_0.23_Pt_1_ on 5 wt % Pd/carbon. Figure 2b shows ED kinetics for a bath selected to give a higher Cu:Pt ratio. In this case, 240 μmol and 80 μmol of Cu^2+^ and PtCl_6_^2−^, respectively, were added over a 60 min period to give a final composition of Cu_1.6_Pt_1_. Interestingly, there was an apparent induction period for deposition of Cu^0^, while Pt was deposited at the outset. The Cu kinetic plot does show, however, that the analyzed Cu^2+^ in the bath agrees very well with the amount of Cu^2+^ syringe-pumped into the bath for the first 20 min interval. Similar experiments were conducted for different bath compositions, and the results are summarized in Table 1. In Table 1, the amounts of Pt and Cu deposited for all samples are shown to illustrate the range of these components in the shell layer. Since the complete deposition of both components did not occur at the deposition conditions that were used, a plot of target deposition vs. actual deposition was constructed and shown in Figure 2c to help select the concentrations to be used for a particular bimetallic shell composition.

### 3.2. Physicochemical Characterization

**Figure 3:**
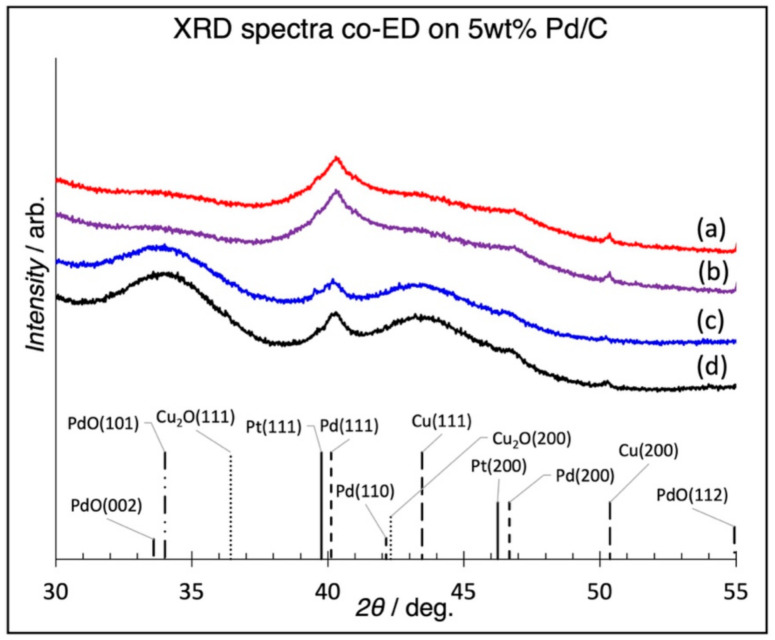
Powder XRD patterns of base Cu_5.8_Pt (**a**), Cu_3.3_Pt (**b**), Cu_0.18_Pt (**c**), and 5 wt % Pd/C catalyst (**d**). The absence of Cu_2_O peaks supports the mixed Pt–Cu shell assignment. The unresolved, broad and diffuse peaks between Pt(111) and Cu(111) are consistent with mixed Cu–Pt alloy shell. The absence of Cu_2_O, Cu^0^, or Pt^0^ peaks indicates no formation of these species and that phase segregation had not occurred.

XRD patterns of selected samples are shown in Figure 3. A Rigaku Miniflex XRD with an ultra-high sensitivity D/tex Ultra 250 1D silicon strip detector has sufficient sensitivity to determine the existence of crystalline structures at these low weight loadings, as evidenced from previous studies [36,37]. For all samples, neither Cu_2_O peaks nor sharp Pt peaks indicative of larger Pt particles were detected, consistent with the assumption that the shell is a uniformly mixed alloy and that segregation into discrete Pt and Cu particles had not occurred. A broad peak does exist in the region between the (111) planes of Pt and Cu, and according to Vegard’s law, the lattice parameter of a solution-phase alloy is a weighted composition of the individual constituents’ lattice parameters. Thus, the peak lies in the expected region for a Cu–Pt shell [38]. Additionally, the peak broadening suggests very small domain sizes, in accordance with the maximum of ~5 ML shell metals deposited by continuous co-ED (Table 1). Shell thicknesses have been previously determined in our laboratory by Scherrer peak broadening for Pt@Pt_2_O_3_ species using this same technique; the diffuse broad peaks are an indication that the shell is, indeed, of mixed Cu–Pt character [39]. Additionally, the XRD patterns show that the base Pd peaks neither shift position (indicating no alloy formation with Pt or Cu according to Vegard’s law) nor change in shape (indicating no growth of the Pd core due to alloying). Thus, we can conclude that the base Pd/C catalyst is substantially unchanged by the co-ED procedure. A broad peak at 2*θ* = 34° in Figure 3c,d could be attributed to PdO. This peak decreases in intensity in Figure 3a,b. Oxidation of Pt nanoparticles supported on carbon under ambient conditions was shown by Banerjee et al. as a shell of Pt oxides over a core of Pt [39]. Given that the oxidation potential of Pd is greater than Pt, it is not unexpected to see surface oxidation [40]. PdO is readily reduced by N_2_H_4_ in the ED bath. Re-oxidation of exposed Pd post-ED could be one explanation for the reappearance of PdO in 3c.

**Figure 4:**
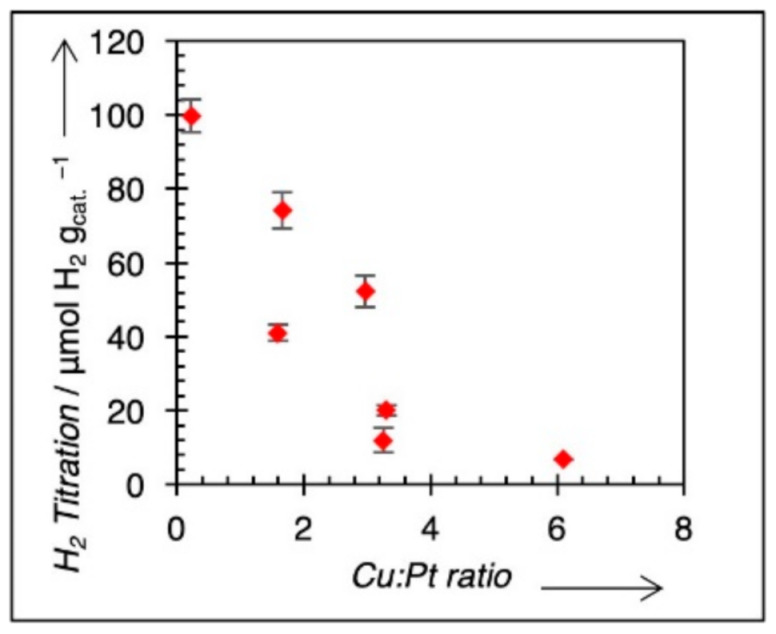
H_2_ titration results for O-pre-covered catalysts. Values decline as Pt is diluted by Cu on the Pt–Cu surface.

Since Pt is the active site for methanol electrooxidation, it is helpful to determine the concentration of Pt surface in the samples using methods other than the electrochemical surface area. To determine the amount of exposed Pt, H_2_ titration of O-pre-covered Pt was used [33,34]. Cu^0^ is not active for H_2_ titration at ambient conditions. Higher heating temperatures were not used to intentionally minimize the potential of Pt and Cu de-alloying in the shell and to prevent the high-temperature formation of Pt–Pd alloys (from the core). Pt and Pd readily form solid solutions across all compositions at elevated temperatures [41,42]. Ternary Pt–Cu–Pd alloys can potentially form at high-temperature; therefore, no high-temperature treatment was used to preserve the Cu–Pt shell/Pd core morphology [22,23,24,25]. The results from H_2_ titration are shown in Figure 4. The uptake of H_2_ decreases with higher Cu/Pt ratios as expected since surface Pt becomes more diluted by Cu, and Cu–O requires elevated temperatures for chemisorption. We also assume that no Pd sites are exposed for the Cu–Pt alloy, which may, in fact, not be the case for some compositions. If surface Pd is present, it will also be active for H_2_ titration. In these cases, the Pt surface site concentration would be over-stated, and methanol oxidation activities relative to Pt surface areas would be under-calculated. Regardless, active site concentrations based on chemisorption are a way to standardize specific activities, which are typically done in conventional catalysis [43,44]. Additionally, there is good agreement in the literature between Pt surface area calculated by chemisorption methods and by H-stripping electrochemical methods [45,46].

**Figure 5:**
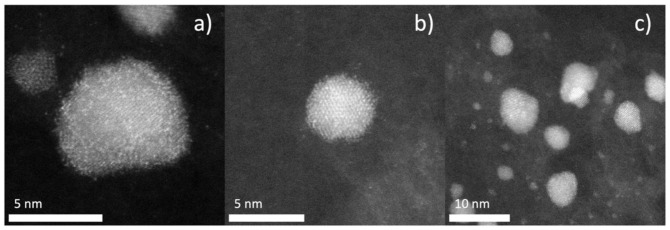
TEM images of Cu_3.3_Pt_1_ sample. *Z*-contrast imaging shows Pt as bright white spots, with Cu shown in darkest gray. The speckling of white spots in (**a**,**b**) show a uniform distribution of Pt in the shell, lattice fringes arise from ordered Pd core (d-spacing calculated from lattice fringes in images (**b**,**c**) equal 0.23 nm, corresponding to known d-spacing of Pd (111) diffraction, 0.223 nm) Uniform core-shell formation can be seen in image (**c**) showing even speckling across several particles.

Results from STEM *Z*-contrast imaging show a uniform distribution of Pt (individual bright spots) over a Pd core. Pd is medium gray, and Cu is dark gray in *Z*-contrast. The distribution of Pt in the shell looks to be both uniform and random. Figure 5a,b shows representative particles. Lattice fringes are present in both images, indicating the ordered Pd core, with a halo surrounding the core containing both bright specks, Pt, and dark gray, Cu. Lattice fringes were analyzed with FFT in ImageJ software, and d-spacing was measured to be 0.23 nm [47]. The known d-spacing for Pd(111) is 0.223 nm; this corroborates the statement that these lattice fringes arise from the Pd core. Figure 5c shows uniform speckling on several particles, indicating that co-ED produces uniform shells of randomly distributed Cu and Pt over Pd cores and that 5a and 5b are representative particles for the overall sample.

### 3.3. Electrochemical Characterization

**Figure 6:**
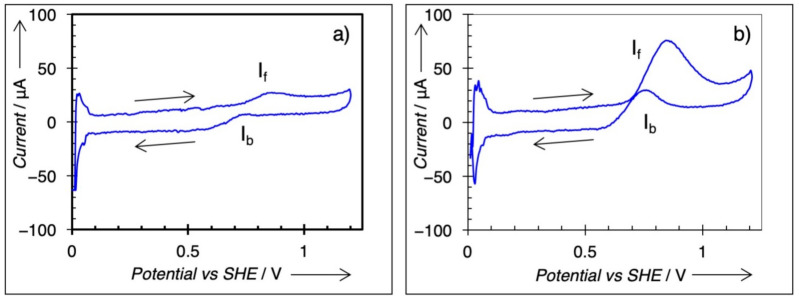
Cyclic voltammetry CV) traces for (**a**) Cu_0.23_Pt and (**b**) Cu_1__.6_Pt. Forward scan peak current (I_f_) at 0.85 V used for comparison of catalysts. Cyclic voltammetry in 0.5 M H_2_SO_4_, 1 M methanol, scan rate 5 mV/s, at 25 °C.

**Figure 7:**
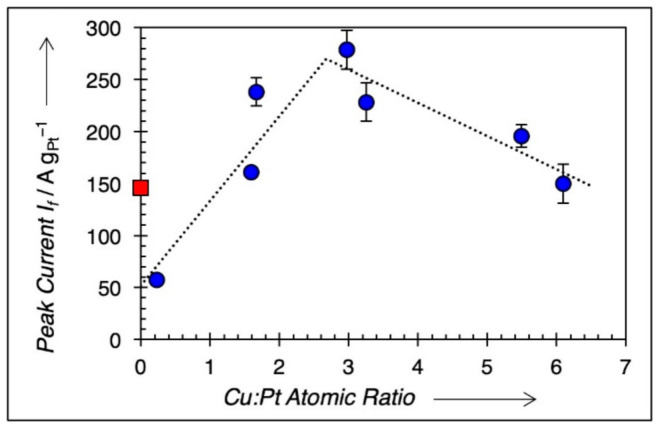
Mass activity of Pd@Cu–Pt/C catalysts prepared by continuous co-ED versus the atomic ratio of Cu and Pt in the shell. Dashed lines are linear regressions of the points to the left and right of peak (peak inclusive), added as a guide to the eye. Red square is a commercial 20 wt % Pt/XC-72 catalyst for comparison.

Cyclic voltammetry was conducted on all samples, and the results are included in Table 2. Each voltammogram shows two remarkable anodic peaks, one in the forward scan (I_f_) and the other in the backward scan (I_b_). The forward current, I_f_ at ~0.85 V, normalized to the mass of Pt (mass activity), was measured as the marker for MeOH electrooxidation activity, in accordance with prior literature [11,12]. Figure 6a,b shows two CV traces for two samples, Cu_0.23_Pt_1_ and Cu_1.6_Pt_1_, respectively. The forward peak current (I_f_) and the backward peak current (I_b_) were marked in Figure 6. Only the forward current (I_f_) was used to determine the mass activity for MeOH electrooxidation in this study. According to Chung et al. [3], the backward oxidation (I_b_) is not affected by the forward reaction; as a result, I_b_ cannot come from a forward scan intermediate. It was debated that in the backward direction, the surface is covered with Pt oxide, thus making I_b_ representative of reduction of a PtO*_x_* surface, which must be stripped before methanol decomposition can occur [3]. Figure 7 shows mass activities plotted against shell composition for different Cu:Pt ratios. A maximum mass activity value is clearly observed at Cu:Pt = 3.0.

## 4. Discussion

The experimental results confirm that a shell composition of Cu:Pt ~3:1 is optimum for MeOH electrooxidation. There are two likely explanations. One explanation is that the addition of Cu to the shell modifies the electronic structure of Pt, specifically the d-band shape and location to the Fermi level, which can affect the strength of adsorption of intermediates, such as CO on the Pt surface. Both computational and experimental data have shown that CO adsorbs less strongly on Pt as the d-band center is shifted away from the Fermi level [8,9]. Both Pt and Cu exist as fcc metals but have different lattice parameters due to their different atomic radii in the fcc lattice. For the situation where Pt exists as an epitaxial overlayer on Cu, the first few Pt lattice layers undergo compression; the Pt–Pt and Cu–Cu bond distances, in bulk metal, are 2.77 Å and 2.56 Å, respectively. Experimentally, it has been shown that the more compressed the Pt overlayer, the weaker the binding energy of CO [8]. It is possible that the addition of Cu along with Pt in our bimetallic shell may function the same way, but not as a discrete epitaxial layer of Pt on Cu since both metals were simultaneously deposited, but as a true mixed Pt–Cu alloy. Intuitively, the expectation is that the optimum composition would be Cu:Pt = 1:1 if this was the mechanism.

Second, the same is true if bifunctionality is the explanation for higher activities. In prior work for the ED of Ru on a commercial 20 wt % Pt/XC-72 catalyst, Ru–Pt catalysts with the loading of Ru on Pt = 0.5 ML were the optimum coverage of Ru [12]. This is consistent with a bifunctional and bimetallic Ru–Pt site where methanol is oxidized on Pt and a proximal Ru site activate H_2_O to form Ru–OH to help remove CO as CO_2_ (Equations (1)–(3) in this manuscript), which is supported by the disordered bimetallic alloy for the Ru–Pt system described by Watanabe [11]. A simple statistical model states the maximum number of Pt-Ru bonds exists at Pt:Ru = 1:1. For the Cu–Pt system, however, the maximum activity occurs at Cu:Pt = 3:1. In previous work by Rossmeisl, adlayers of Cu on a Pt surface performed best at a preparation giving ϴ_Cu_ = 0.5 ML, despite the computational models predicting optimum performance at Cu_3_Pt; the authors attributed this to possible surface rearrangement to form Cu_3_ trimers (Cu_3,tri_) [5]. Other computational results have suggested that OH adsorption to Cu is most stable in the three-fold hollow made by Cu_3,tri_, as opposed to linear Cu–OH adsorption [21].

**Figure 8:**
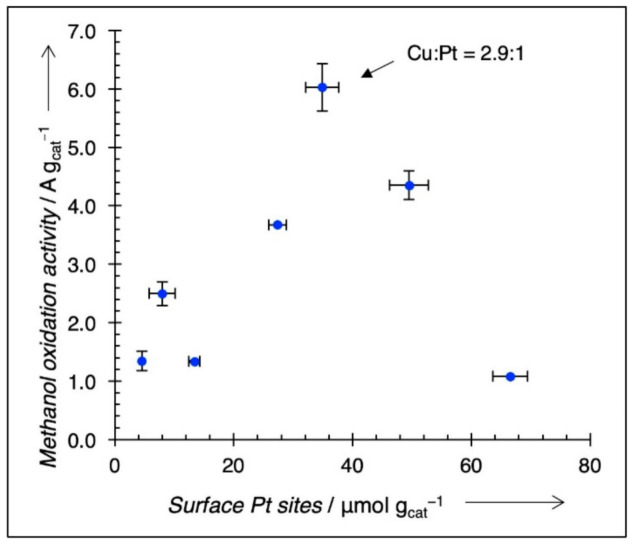
MeOH electrooxidation activity vs. a number of Pt surface sites (Pt_s_). If increased activity is simply because there are more Pt surface sites, there should be an upward linear trend. However, this is not the case, and maximum activity is maintained at Cu_3_Pt_1_.

To discount the possibility that increased activity is simply due to a higher number of surface Pt sites (Pt_s_), mass normalized I_f_ (A/g cat) is plotted against Pt surface sites (μmol Pts/g cat) for each catalyst in Figure 8; the Pt_s_ values are taken from Table 1. The maximum activity is preserved at the Cu:Pt ratio ~3:1, and there is no clear positive trend relating Pt_s_/g cat to mass activity, indicating that simply increasing the number of Pt_s_ does not account for the activity trends for among the catalysts prepared by continuous co-ED. We can now assume that the formation of Cu_3,tri_–Pt ensembles are the preferred sites for MeOH electrooxidation and that the concentration of these Cu_3,tri_–Pt pairs is highest for Cu_3_Pt. There are also some Cu_3,tri_ sites at different Cu:Pt ratios, which were also synthesized using co-ED. To determine the concentration of these sites, a simple model is used.

**Figure 9:**
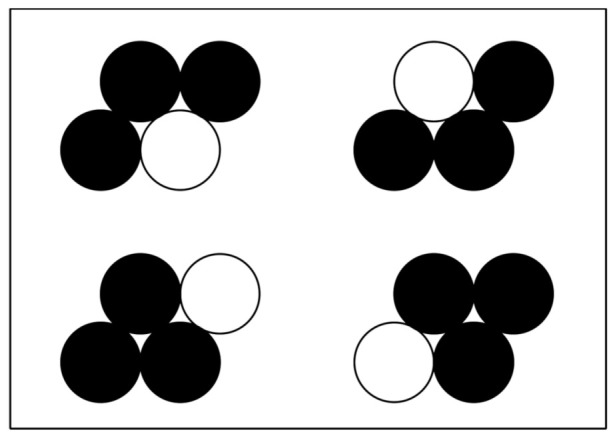
Positions for deposition of three Cu atoms (black) in a four atom fcc primitive cell. Only the bottom two structures have the single Pt atom (white) adjacent to a Cu_3,tri_ with three-fold hollow.

**Figure 10:**
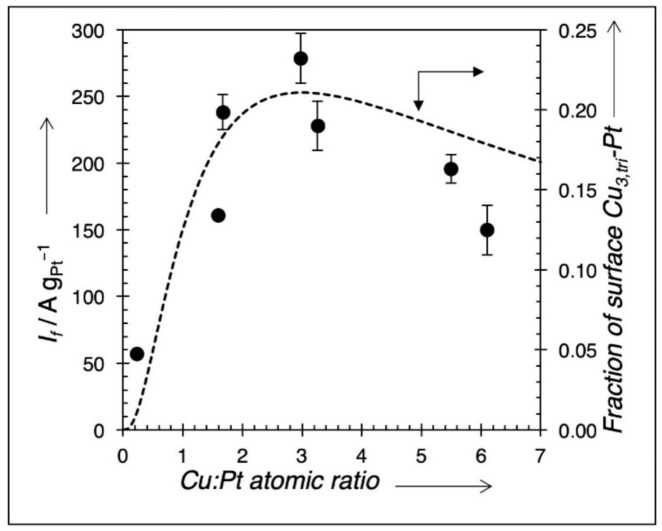
Calculated fraction (dashed line) of surface composed of Cu_3,tri_–Pt pairs versus Cu:Pt ratio in shell overlaid with Pt mass activity for CH_3_OH electrooxidation.

The unit cell for the surface of an fcc metal can be divided into a series of two-dimensional, four-atom primitive cells arranged in a parallelogram shape. If we assume random packing of deposited Cu and Pt atoms in the four-atom parallelogram, a simple probability for the population of these sites as independently occurring events can be used. The likelihood of depositing Cu or Pt at a lattice point is directly related to the ratio of the metals being deposited, or P_Cu_ = X_Cu_ = N_Cu_/(N_Cu_ + N_Pt_), where P_Cu_ is the probability of Cu being deposited, X_Cu_ is the atomic mole fraction of Cu being deposited, and N_Cu_ and N_Pt_ represent the molar amounts of Cu and Pt, respectively, being deposited; likewise, P_Pt_ = X_Pt_ = N_Pt_/(N_Cu_ + N_Pt_). There are 16 unique arrangements where 0, 1, 2, 3, or 4 Cu atoms can be placed in the primitive cell; four of these structures contain three Cu atoms, but only two of them have a Cu_3,tri_ structure adjacent to a Pt atom, as shown in Figure 9, a graphical representation of this model. The probability of depositing 3 Cu atoms and 1 Pt atom in this primitive cell in the proper configuration is P(Cu_3,tri_–Pt) = 2 • P_Cu_^3^ • P_Pt_. Substituting for an atomic fraction of Cu gives P(Cu_3,tri_–Pt) = 2 • X_Cu_^3^ • (1−X_Cu_). If we define r as the ratio of N_Cu_/N_Pt_ = X_Cu_/X_Pt_ and rearrange, the equation gives the probability P(Cu_3,tri_–Pt) = 2 • r^3^/(1 + r)^4^. A plot of this probability for different ratios of Cu:Pt is shown in Figure 10 overlaid with the experimental mass activity results. Using I_f_ = *n*Fk_f_C_O_ where *n* is a number of electrons removed during oxidation, k_f_ is a forward reaction (oxidation) constant, F is Faraday’s constant, and C_O_ is the concentration of adsorbed methanol, then C_O_ ∝ X(Cu_3,tri_–Pt) [48], which is proportional to the number of Cu_3,tri_–Pt pairs. The shape of the curve for mass activity vs. calculated fraction of Cu_3,tri_–Pt pairs are in good agreement, although there is some deviation at high Cu:Pt ratios. Using the same justification as Watanabe, the bifunctional mechanism of the Cu_3 tri_–Pt site for MeOH electrooxidation is warranted [11]. Since codeposition of Cu and Pt on the Pd, core takes place at 25 °C, and there is no high-temperature annealing after preparation of the catalyst, we can also logically assume that the positions of Cu and Pt in the fcc lattice are random (as shown by STEM images, in Figure 5) and stable during electrooxidation. In summary, the method of continuous co-ED of both Pt and Cu components in specific ratios and in a controlled manner has resulted in the synthesis of advanced, direct methanol fuel cell catalysts. It also has provided one of the few cases where direct comparisons can be made to test the predictive capabilities of computation-based studies for this important reaction.

## 5. Conclusions

Continuous co-ED provides a way to make shells of mixed-metal composition with highly controlled ratios of constituent metals. This technique was successfully applied for the creation of methanol electrooxidation catalysts for DMFC applications. Mixed shells of Cu–Pt has shown markedly higher Pt mass activities than a commercial 20 wt % Pt/XC-72 electrocatalyst. The enhanced activity was correlated with the formation of bifunctional Cu–Pt sites predicted from previous computational studies [5]. The bifunctional site provides close proximity of OH_ads_ and CO_ads_ to promote facile oxidation to CO_2_, thus lowering the strong inhibition effect of strongly adsorbed CO on Pt sites. Further, the ability of co-ED to prepare a wide series of bimetallic Cu–Pt shells has permitted correlation of experimental results with the optimum Cu:Pt = 3:1 ratio predicted by others [5,6]. A relatively simple statistical model has shown that Cu_3,tri_–Pt pair sites are likely to be the specific sites for enhanced activity and that the concentration of these species control activity. In these cases, H_2_O is preferentially adsorbed in the three-fold hollow of a Cu_3_ trimer, which is adjacent to a Pt site containing strongly adsorbed CO; the reaction is then facilitated to form CO_2,_ which readily desorbs. Activity trends correlate quite strongly with the calculated surface fraction of Cu_3,tri_–Pt pairs.

Of economic importance is the more efficient use of Pt with this method of preparation. Pt is used only in a thin shell layer, improving the effective dispersion of Pt compared to bulk Pt alone. In this study, Pt weight loadings varied between 0.9 and 2.4 wt %. Although the core catalyst used in this study was a commercial 5 wt % Pd/C, there is no fundamental reason the core metal could not be at a lower concentration and/or a less expensive metal, so long it is stable under reaction conditions.

Further application of this method of preparation to other metals, which have OH adsorption at 3-fold hollows in an fcc lattice, could show similar mass activity trends. Currently, studies are being made with Ni–Pt and Co–Pt shells prepared using co-electroless deposition [49]. Clearly, less expensive, more active, and more stable electrocatalysts will be critical for the commercialization of any fuel cell technology.

## 6. Patents

J.R. Monnier, G.L. Tate, W. Xiong, and B.H. Meekins, “CO-ELECTROLESS DEPOSITION METHODS FOR FORMATION OF METHANOL FUEL CELL CATALYSTS, US pat. Appl. US 2020/0313214, 1 October 2020.

## Figures and Tables

**Table 1:** Summary of catalysts prepared and tested with cyclic voltammetry. One ML is defined as the number of atoms required to cover the surface of the Pd core. The surface concentration of the Pd core was determined by H-titration of O-pre-covered Pd. Weight loadings of Pt and Cu refer to weight loadings of each metal added to the 5% Pd/C. The Pd loading was not factored into the Pt and Cu loadings. The concentration of Pt surface sites was determined from the H_2_ titration of O-pre-covered Pt sites.

Empirical Formula	Pt (wt %)	Cu (wt %)	ML (Cu + Pt)	Number of Pt_s_ (10^18^ Sites × g^−1^ Cat)
Cu_0.2_Pt_1_	1.9	0.14	1.2	40
Cu_1.6_Pt_1_	2.4	1.2	3.1	17
Cu_1.7_Pt_1_	1.9	1.0	2.5	30
Cu_3.0_Pt_1_	2.3	2.2	4.5	21
Cu_3.3_Pt_1_	1.1	1.2	2.4	8.1
Cu_5.5_Pt_1_	0.90	1.8	3.3	4.8
Cu_6.1_Pt_1_	1.1	2.0	3.7	2.7

**Table 2:** Summary of CV results.

Empirical Formula	Peak Current (A × g^−1^ Pt)
Pt (commercial)	146
Cu_0.2_Pt_1_	57
Cu_1.6_Pt_1_	161
Cu_1.7_Pt_1_	238
Cu_3.0_Pt_1_	278
Cu_3.3_Pt_1_	228
Cu_5.5_Pt_1_	296
Cu_6.1_Pt_1_	150

## Data Availability

The data presented in this study are available on request from the corresponding author.
